# Structural and biochemical insight into allosteric regulation of the human 2-aminoadipic semialdehyde synthase, a bifunctional enzyme involved in lysine catabolism

**DOI:** 10.21203/rs.3.rs-9509715/v2

**Published:** 2026-05-26

**Authors:** Ruoxi Wu, Susmita Khamrui, Tetyana Dodatko, Yufei Xiang, Afrooz Golestanian, Ngoc Dung Pham, Kunal Kumar, Yi Shi, Roman Osman, Robert J. DeVita, Sander M. Houten, Michael B. Lazarus

**Affiliations:** 1 Department of Pharmacological Sciences, Icahn School of Medicine at Mount Sinai, New York, NY 10029, USA; 2 Drug Discovery Institute, Icahn School of Medicine at Mount Sinai, New York, NY 10029, USA; 3 Department of Genetics and Genomic Sciences, Icahn School of Medicine at Mount Sinai, New York, NY 10029, USA

## Abstract

Multiple clinically significant inborn errors of metabolism occur in the lysine degradation, even so the regulation of this biochemical pathway remains understudied. The initial rate-limiting step of lysine catabolism is catalyzed by the bifunctional enzyme 2-aminoadipic semialdehyde synthase (AASS). Therefore, understanding the regulation of AASS activity in normal and disease states will be critical for developing novel therapeutic approaches that modulate lysine degradation flux. Here, we report the cryo-EM structure of the full-length 400 kDa tetrameric AASS enzyme complex with substrates and products bound to both the lysine-2-oxoglutarate (LOR) and saccharopine dehydrogenase (SDH) catalytic domains. Our full-length structure shows that two SDH dimers are connected by a long alpha-helix group and flexible loops to a core LOR tetramer. This functional arrangement gives the SDH domain flexibility to move and readily access the products from the LOR domain. Through chemical screens, we also identified allosteric compounds that modulate activity of the AASS. Maleimides, a class of Michael-Addition chemistry substrates, activate the enzyme by reacting with cysteine 414 and shifting the pH optimum for biochemical activity. This observation can be explained mechanistically by pronounced changes in the affinity for NADPH as a function of pH. Remarkably, the mutagenesis of this allosteric Cys414 has dramatic effects on the enzyme’s conformation and activity despite being more than 12 Å from the active site. Simulations also show how changes at this distal site can affect the dynamics of the enzyme. Another compound, 1-methylnicotinamide, inhibits the enzyme by catalyzing the formation of a disulfide bond, which was observed through cryo-EM structural studies. Combined, our data indicate that AASS can be modulated at multiple allosteric sites through small molecules providing opportunities to regulate lysine catabolism by either inhibition or activation. Moreover, small changes near the hinge of an enzyme can have large effects on the catalytic properties of the enzyme. Lastly, targeting allosteric cysteines may be a general strategy for modulating enzyme activity by altering the dynamics of the enzyme.

## Introduction

**Figure 1.**
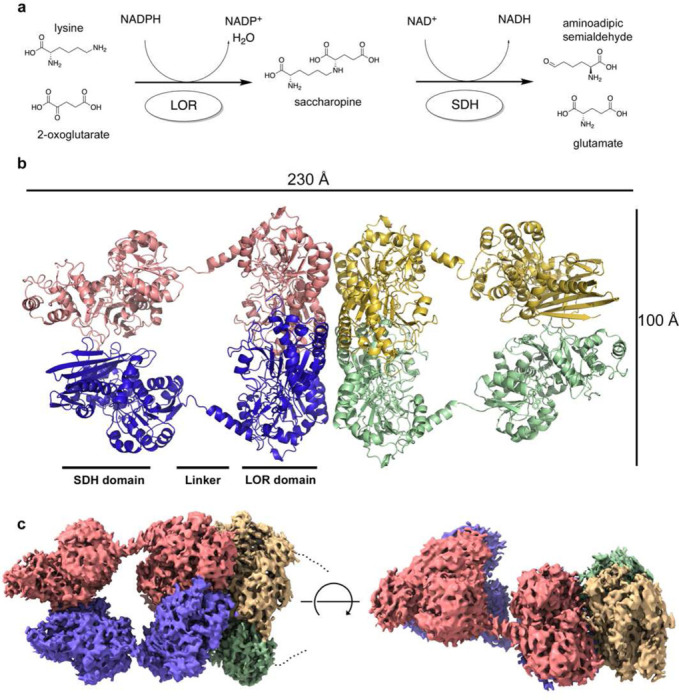
Overall structure and function of AASS. (A) Enzyme reaction of LOR and SDH domains of AASS. (B) Overall structure of AASS tetrameric complex. The complex is colored by chain, with the two domains indicated along with the helical linker connecting them. (C) High-resolution structure of the tetrameric complex, with 6 of the 8 domains visible. The electron density is shown colored by the corresponding chains and rotated to show two different perspectives.

2-Aminoadipic acid semialdehyde synthase (AASS), which catalyzes the first two steps in lysine degradation via the saccharopine pathway, has emerged as an attractive target to treat glutaric aciduria type 1 (GA1) and pyridoxine-dependent epilepsy (PDE-ALDH7A1). AASS is a bifunctional enzyme with an N-terminal lysine-2-oxoglutarate reductase (LOR) domain and a C-terminal saccharopine dehydrogenase (SDH) domain (Figure 1A). The LOR domain catalyzes the reductive amination of L-lysine and 2-oxoglutarate into saccharopine (EC 1.5.1.8), which constitutes the first committed and rate-limiting step in lysine degradation. The subsequent oxidative deamination of saccharopine into 2-aminoapidic semialdehyde and glutamate by the SDH domain completes the e-deamination of lysine (EC 1.5.1.9). Crystal structures have been reported for the isolated LOR and SDH protein including human, but a structure of the bifunctional enzyme complex did not exist.

Lysine degradation is of considerable clinical interest. GA1 and PDE-ALDH7A1 are neurometabolic diseases, both with significant unmet medical need lacking FDA approved treatment. These disorders are caused by defects in glutaryl-CoA dehydrogenase (GCDH) and antiquitin (ALDH7A1), respectively, which are both enzymes that function downstream of AASS in lysine degradation leading to build up of toxic metabolites. We and others have hypothesized that GA1 and PDE-ALDH7A1 can be treated through pharmacological inhibition of AASS, which will lead to reduction in the accumulation of the toxic substrates [1–5]. AASS is of particular interest, because its deficiency due to AASS mutation leads to a biochemical abnormality without clinical significance [6, 7]. This suggest that inhibition of AASS and specifically of the LOR domain is potentially safe. Accordingly, therapeutic developments using small molecules and genetic approaches to target AASS have been reported [1, 3, 8–13].

Lysine is an essential amino acid, but very few studies have addressed lysine homeostasis. Increased understanding of the regulation of lysine degradation may lead to the development of novel approaches that limit the accumulation of toxic substrates in GA1 and PDE-ALDH7A1. It is known that *AASS* expression is controlled by the glucocorticoid receptor (GR) and Krüppel-like factor 15 (KLF15) [14, 15], but post-transcriptional and allosteric regulatory mechanisms have not been reported yet. To aid in understanding the molecular mechanisms of AASS regulation, we now report multiple cryo-electron microscopy (cryo-EM) structures of the full-length AASS in complex with its natural substrates and products NADPH, NADH and saccharopine. We also elucidate mechanisms underlying allosteric activation and inhibition of the LOR enzyme.

## Materials and Methods

### Materials

NADPH, tetrasodium salt was from Roche or MilliporeSigma. 1-(2-hydroxyethyl)-2,5-dihydro-1H-pyrrole-2,5-dione (**M4**), 1-butyl-2,5-dihydro-1H-pyrrole-2,5-dione (**M7**) and 1-ethyl-2,5-dihydro-1H-pyrrole-2,5-dione (NEM, **M1**) were from Aaron Chemicals LLC and purchased through MolPort, Inc. 1-butyl-3,4-dimethyl-2,5-dihydro-1H-pyrrole-2,5-dione (**M8**), 3-bromo-1-phenyl-2,5-dihydro-1H-pyrrole-2,5-dione (**M9**), 1-(3,5-dimethylphenyl)-2,5-dihydro-1H-pyrrole-2,5-dione (**M5**), 1-(2-fluorophenyl)-2,5-dihydro-1H-pyrrole-2,5-dione (**M10**), 1-[3-(trifluoromethyl)phenyl]-2,5-dihydro-1H-pyrrole-2,5-dione (**M11**) were from Vitas M Chemical Limited and purchased through MolPort, Inc. 1-phenyl-2,5-dihydro-1H-pyrrole-2,5-dione (**M2**), 1-*tert*-butyl-2,5-dihydro-1H-pyrrole-2,5-dione (**M3**), 1-methyl-2,5-dihydro-1H-pyrrole-2,5-dione (**M6**), trigonelline HCl, niacin, nicotinamide and N-methyl-2-pyridone-5-carboxamide were from MilliporeSigma. N-(2-aminoethyl)maleimide hydrochloride (**M12**) and 3-maleimidopropionic acid (**M13**) were from TCI. 1-methyl-1,4-dihydronicotinamide and 1-methylnicotinamide (chloride) were from Cayman Chemical Company.

### Gene cloning, protein expression and purification

For enzyme assays, sumostar-LOR (residues 21–470) was purified from *E. coli* as previously described [1]. For thermal shift experiments, sumo-LOR protein was purified from *E. coli* followed by sumo tag cleavage as previously described [1]. For full-length protein for structural studies, the full-length gene (Origene) was cloned into a modified pFastBac HTA vector containing an N-terminal twin strep tag, followed by 10X His tag followed by HRV3C cleavage site followed by the AASS gene (encoding residues 21–926). Bacmid DNA was generated in DH10Bac cells (Invitrogen) and protein was expressed in Sf9 cells (Expression Systems) using the Bac-to-Bac baculovirus expression system (Invitrogen). In brief, ~2μg recombinant bacmid DNA and 4 μl FuGENE HD transfection reagent (Promega) in 100 μl transfection media (Expression Systems) were added to 500,000 Sf9 cells plated in 500 μl of SF921 media in wells of a 12-well plate. After 5 days at 27°C, the supernatant was collected as P0 viral stock, P1 and P2 baculovirus was obtained by adding P0 viral stock to the cells at a density of 2 × 10^6^ cells per mL while shaking at 27°C. Expression of AASS full-length for structural studies was carried out by infection of Sf9 cells at a cell density of 2–3 × 10^6^ cells per mL with P2 virus at 1:100 ratio. Cells were collected by centrifugation at 48 hours postinfection and stored at −80°C until use.

The insect cells were resuspended with 150mM NaCl, 20mM HEPES pH7.5 and 50mM imidazole (buffer A) plus 10% glycerol and homemade protease inhibitor cocktail (500 μM AEBSF, 1 μM E-64, 1 μM leupeptin, 150 nM aprotinin). Then, the cells were sonicated (Qsonica) for 12 minutes and centrifuged at 36,000 x g for 30 minutes. The supernatant was loaded onto Ni-NTA resin (0.5 mL/L culture, Qiagen) that was pre-equilibrated with buffer A. After 1 h incubation, the resin was washed with the same buffer and eluted with 150mM NaCl, 20mM HEPES pH7.5 plus 250 mM imidazole. After overnight cleavage by 3C protease, the target protein was loaded onto a Superdex 200 10/300 Increase Column (GE) in 150mM NaCl, 20mM HEPES pH7.5, and 0.5mM TCEP.

### Electron microscopy

For electron microscopy, 3μl of AASS full-length at a concentration of 2.5 mg/mL was applied to glow-discharged Quantifoil holey carbon grids (Cu, R1.2/1.3, 300 mesh). The grids were blotted for 2 seconds and plunged into liquid ethane with a Vitrobot plunger (4°C and 90% humidity). Cryo-EM data were collected with a Titan Krios microscope (FEI) operated at 300 kV and images were collected at a nominal magnification of 165,000 corresponding to a pixel size of 0.73Å with a defocus range of −0.6 to −2 μm. The images were recorded on a Falcon IV direct detector in super-resolution mode at the end of a GIF-Quantum energy filter operated with a slit width of 10–20 eV. A dose rate of 4.4 electrons per pixel per second and an exposure time of 6 seconds were used, resulting in an accumulated total dose of 50 electrons per Å^2^.

### Image processing

Movies were motion-corrected using MotionCor2 [16] and imported to cryoSPARC [17] for further processing. Contrast transfer functions were estimated using patchCTF in cryoSPARC. An initial model was produced from a subset of micrographs using blob picking, followed by multiple runs of extraction, 2D classification, selection of key classes and generation of a model ab initio. Subsequent map models were produced from a curated micrograph set using particles found by picking using the initial map model as a template. Particles were extracted, subjected to 2D classification and a final particle stack was obtained by iterative rounds of 3D classification generating several bad models from rejected particles as a sink in hetero-refinement. A final map was obtained using NU-refinement [18]. Structures were built by docking using Phenix [19] or manually in Coot [20] based on previous crystal structures of AASS LOR domain [1] and SDH domain (PDB 5L78) and further refined using PHENIX with manual adjustments in Coot. Final maps were imported to PyMOL [21] and UCSF Chimera [22] for generating figures shown in the manuscript.

### Differential Scanning Fluorimetry

The thermal shift assay to assess the effect of pH for the LOR domain was performed on QuantStudio 3 real-time PCR system (Applied Biosystems). Purified proteins of 4 mg/mL stock concentration was diluted to a final concentration of 0.6 mg/mL in 50 mM potassium phosphate of pH 7 and pH 8, respectively containing 150 mM NaCl. 2.5 L of 8X SYPRO orange (Applied Biosystems) was added in a final 20 L reaction volume. This 8X SYPRO orange was prepared by diluting the manufacturer supplied dye 250-fold in H_2_O. The reactions were kept at 25°C for 2 min and then heated from 25°C to 95°C with a rate of 0.05°C/S. The change in the fluorescence intensities of SYPRO orange was monitored as a function of the temperature and analyzed by Protein Thermal Shift Software 1.3. Each reaction was performed in 4 replicates.

### Metabolite screen

We screened the Human Endogenous Metabolite Compound Library purchased from MedChemExpress (HY-L030). The library contained a total of 980 compounds distributed over 4 384-well plates. The 738 compounds in DMSO were distributed over 3 plates, the remaining 233 compounds in water and 9 in ethanol were on a fourth plate. Columns 1, 2, 23 and 24 were filled with solvent (DMSO for plates 120133 or water for plate 4). Most compound concentrations were 10 mM, some were 2 mM (supplemental table). For screening, each well of a 384-well plates first received 25 μL reagent containing 100 mM Hepes pH 7.4, 0.6 mM NADPH, 30 mM L-lysine, 0.5% Triton X-100 and 0.36 μg/mL recombinant LOR. After centrifugation of the plate, 0.5 μL of the compounds was transferred to the wells (final 100x dilution, most compounds are at final concentration of 100 μM). After an incubation of 10 minutes, the enzyme reaction was started by adding 25 μL of 2 mM 2-oxoglutarate to the wells. The wells that served as positive control (PC) for inhibition (columns 1 and 24) received 25 μL water. Wells A2 and B2 received 0.5 μL of a compound solution previously demonstrated to activate LOR to serve as PC for activation. Plates were then centrifuged and absorbance measured at 340 nm. Four sequential reads approximately every 20 minutes were performed which allowed the calculation of decrease in absorption due to NADPH consumption (ΔA). In contrast to the analysis of a single endpoint, the decrease in absorption allows to assess false positives due to absorption of the screening compounds. For each plate, we calculated the Z’-factor for the 3 sequential ΔA values, a measure of the statistical effect size that serves as an indicator of the quality and performance of the assay in HTS. An assay for which the Z’-factor is 1 > Z’ ≥ 0.5 is considered excellent for screening. The average Z’-factor in our screen was 0.69 (minimum 0.55 and maximum 0.75). For each compound, we calculated a z-score, which is the number of SDs that this well deviates from all other experimental wells on the plate. For each compound, we also calculated a % activity, which reflects activity compared the average of the negative controls (NC). The NC were calculated from wells in column 2 and 23, which only contained the solvent. Final analysis was performed on the data from the first 2 measurements. In general, the z-score and % activity had consistent results.

### Mass Spectrometry

Protein samples were reduced in 8 M urea containing 5 mM TCEP and DTT at 57 °C for 1 hour, followed by alkylation with 50 mM iodoacetamide (IAA) for 30 minutes at room temperature in the dark. The alkylated samples were then diluted 8-fold with digestion buffer (50 mM ammonium bicarbonate, 2% acetonitrile (ACN) in LC/MS-grade water). Samples were then digested with Glu-C protease at a 1:50 (w/w) enzyme-to-substrate ratio at 37 °C overnight. An additional aliquot of Glu-C (1:50 w/w) was added, and digestion was continued for another 4 hours at 37 °C. Following proteolysis, peptide mixtures were acidified with formic acid (FA), desalted using self-packed stage tips, and analyzed using a Vanquish NEO UHPLC system coupled to an Exploris 480 Orbitrap^™^ mass spectrometer (Thermo Fisher). Briefly, desalted peptides were loaded onto a C18 analytical column (1.7 μm particle size, 120 Å pore size, 75 μm × 5 cm; Aurora Rapid, IonOpticks) and separated using a 28-minute LC gradient (3–5% B from 0–2 min, 5–40% B from 2–17 min, 40–80% B from 17–22 min, equilibration at 3% B from 22–28 min; mobile phase A consisted of 0.1% FA in water, and mobile phase B consisted of 0.1% FA in ACN). The flow rate was set to 500 nL/min. The mass spectrometer was operated in data-dependent acquisition mode. The 20 most abundant precursors (mass range 350–2000 m/z; charge states 2–6) were selected for fragmentation by high-energy collisional dissociation. MS1 scans were acquired at a resolution of 120,000, and MS/MS scans at a resolution of 7,500. A quadrupole isolation window of 2 Th and a maximum injection time of 100 ms for MS/MS were applied.

Raw MS data were analyzed using FragPipe (v22.0) with the default workflow settings [23]. For MSFragger search parameters, mass tolerances were set to 10 ppm for MS1 and 20 ppm for MS2. Enzymatic digestion was specified as Glu-C allowing up to two missed cleavages. Variable modifications included: (1) +57.02146 Da (carbamidomethylation by IAA) or +125.0476 Da (NEM modification) on cysteine residues; (2) +15.9949 Da (oxidation) on methionine; (3) +42.0106 Da (acetylation) at the protein N-terminus. Peptide-spectrum matches were manually verified. MS1 abundances of modified peptides across samples were quantified by extracting the area under the curve (AUC) using Xcalibur software.

Modification efficiency for each cysteine residue was calculated as the ratio of the AUC of the NEM-modified peptide to the total AUC of both NEM- and IAA-modified peptides:

$$\frac{AUC\;of\;NEM\;modified\;peptide}{\left(AUC\;of\;NEM\;modified\;peptide+AUC\;of\;IAA\;modified\;pepitide\right)}.$$

If multiple peptides containing the same cysteine residue were identified, the modification efficiency was calculated individually and then averaged to obtain the final value for that cysteine site. The proteomics data have been deposited to the MassIVE repository under accession number MSV000097740

### m-PEG maleimide labeling

m-PEG-Mal, MW 10,000 was from BroadPharm (BP-23711, San Diego, CA). LOR protein was diluted to ~ 0.325 mg/mL in 50 mM KPi pH 7.4, combined with an equal volume m-PEG-mal at 0.5 mg/mL in 50 mM KPi pH 7.4 and then incubated for 30 minutes at room temperature. The reaction was subsequently quenched by adding DTT to a final concentration of 10 mM after which samples were prepared for SDS-PAGE and immunoblotting with 6X His Tag Polyclonal Antibody, DyLight^™^ 680 (Thermofisher). Pretreatment with maleimides was performed for 10 minutes at room temperature before m-PEG-mal labeling.

### Molecular Dynamic Studies of the LOR

We used the structure of the tetramer to obtain a model of the monomer. The missing sequence 331–339 was built based on the AlphaFold structure AF-Q9UDR5. AASS also contains NADPH and saccharopine - the reduction product of the enzymatic reaction. We have designed a force field of the oxidized form of saccharopine (SOR, see below for force field development) and placed it in the position of saccharopine. We conducted our MD simulations on the monomer extracted from the tetramer with the NADPH and SOR. The mutation Cys414Asp of the enzyme was constructed by replacing the Cys with an Asp residue. The NEM and the N-(3,5-dimethyl)phenylmaleimide derivatives of Cys414 were prepared by superimposing the cysteine moieties of the derivatives on Cys414.

The system was prepared for simulations in AMBER24 [24]. A periodic box with boundary at 10 Å from the solute was filled with waters and a neutralizing KCl concentration of 0.15 M. The system was minimized, heated, and equilibrated with positional constraints that diminished from 10 to 0 kcal/mol/Å^2^ over 5 ns. Production runs at NPT conditions were of 1 μs length, saving 100,000 structures of the trajectory.

### Design of Force Fields

#### NADPH :

The structure of NADPH was divided into three residues: dihydronicotinamide riboside (NRP), biphosphate (DPO), and adenosine-2’-phosphate (ARP). The force fields for each of the structures were obtained by structural optimization and derivation of RESP charges with Jaguar [25]. Each of the residues was blocked at the connections with the other residues. After the structural optimization and charge derivation, the blocking groups were removed, and the charges of the remaining residues were adjusted so that the charge of each residue was zero.

#### 2-[[(5S)-5-amino-5-carboxypentylidene]amino]pentanedioic acid (PubChem CID 164083912).

The initial structure was constructed from the saccharopine in the crystal structure. The structure was optimized with Jaguar, and the charges were derived using the RESP procedure.

#### S-(1-Ethyl-2,5-dioxopyrrolidin-3-yl)-L-cysteine (PubChem CID 58071243):

The structure was blocked by an acetyl and an N-methyl group at the termini of the cysteine moiety. The structure was optimized with Jaguar, and the charges were derived using a RESP procedure. After removing the blocking groups, the charges were adjusted similarly to above. The derivative was inserted into the protein by superimposing it on the Cys moiety. The residue is named CYN.

#### S-(1-(3,5-dimethylphenyl)-2,5-dioxopyrrolidin-3-yl)-L-cysteine:

The same procedure was used as the *CID 58071243.* The residue is named CYP. The force fields are available upon request.

### Analysis of simulation results

All analyses were performed with AmberTools25.

The system was separated to the protein and the (NADPH + SOR) as the ligand. MMGBSA analysis was run with igb=8 and the radii of the components were adjusted to mbondi3. The decomposition was run with idecomp=4, to allow the evaluation of the interaction energy between the protein and each of the residues in the combined ligand, as well as between the SOR and the components of the NADPH.

## Results

### Structure of full-length AASS

We previously reported the apo crystal structure of the LOR domain of AASS [1]. There is also a crystal structure of the SDH domain that was deposited without a manuscript (PDB 5L78), but no information is available about how the domains interact or how the LOR domain binds substrates. Therefore, we purified full-length human AASS protein in *sf9* cells and incubated with NADPH, NADH, and saccharopine and collected single-particle cryo-EM data. We first obtained a low-resolution structure map of the full-length protein (Supplementary Figure 1), and a model was built based on the map (Figure 1B and Supplementary Table 1). The full-length structure is tetrameric, with a core tetramer of the LOR domain at the center of the complex and two SDH dimers sticking out on each side. The resolution was poor from this structure because of the flexibility between each SDH dimer and the LOR tetramers (Supplementary Figure 2). From another dataset, a high-resolution view of a substructure containing 6 domains (2 SDH domain and 4 LOR domains, Figure 1C) was obtained. This structure provides more details about how the domains are connected. There is a long alpha-helix that bridges the two domains, and there are flexible linkers on both sides of the alpha helix. Alignment between the full-length AASS and the 6-domain high resolution substructure shows that the SDH dimer from the latter rotates approximately 40 degrees backward from the full length SDH dimer, while the two LOR tetrameric cores align well (Supplementary Figure 3). The long alpha-helix also aligns well, indicating it is the linkers between the two domains that render the flexibility of the full-length structure. This flexible motion could allow the SDH domain to readily access the products from the LOR domain, which will then become its substrates without dissociation out of the enzyme.

### Structure of the individual SDH and LOR domains bound to substrates

**Figure 2.**
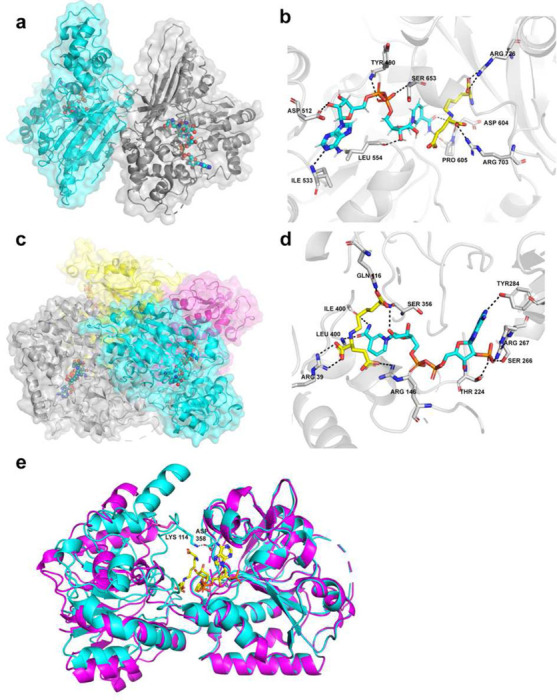
Structure of the individual domains of AASS. (A) Overall structure of the SDH domain, shown as the dimer and colored by chain. NADH and saccharopine are shown as spheres in the active site. (B) Closeup of SDH active site. NADH is shown in blue sticks, saccharopine in yellow. Putative contacts are shown with dashed lines to indicated residues. (C) Overall structure of the LOR domain, shown as the tetramer and colored by chain. NADPH and saccharopine are shown as spheres in the active site. (D) Closeup of LOR active site. NADPH is shown in blue sticks, saccharopine in yellow. Putative contacts are shown with dashed lines to indicated residues. (E) Overlay of apo crystal structure (PDB 8E8U, magenta) with the new cryo-EM complex (blue), with substrates (yellow) and key residues (cyan) shown as sticks.

We were then able to obtain high resolution structures of the SDH domain and the LOR domain alone, using masks to focus on these subsections of the full-complex (Supplementary Figure 1. The SDH domain forms a dimer that is connected by interacting loops at the tip of the domain (Figure 2A). The interface is only 850 Å^2^ [26], yet the dimer interface is identical in all of our structures and to that observed in the crystal structure (5L78), further supporting the SDH dimer itself is rigid despite the motion of the dimer with respect to the AASS complex core. The NADH and saccharopine are bound in cleft of the V-shaped domain, similar to the NAD^+^ in 5L78. NADH and saccharopine are contacted by a series of residues in the active site that bind and orient them: Ile533, Asp512, Tyr490, and Ser653 contact the adenosine diphosphate of NADH through side chain and backbone contacts, whereas Asp604 and Pro605 backbones contact the acetamide portion (Figure 2B). The saccharopine is mainly bound by two arginine residues, Arg703 and Arg726, which make ionic interactions with two of the three carboxylic acids of this substrate, whereas the third carboxylic acid of saccharopine contacts the ribose of NADH.

The LOR domain is tetrameric, also identical to that observed in our crystal structure through symmetry contacts [1], consisting of a dimer of dimers that share a long diagonal surface of 1900 Å^2^ (Figure 2C). The substrates and products bind in each monomer of LOR in the cleft between the two lobes, with clear density for the substrates (Supplementary Figure 4). NADPH and saccharopine are bound by a series of contacts in the LOR active site (Figure 2D). The saccharopine is positioned just over the nicotinamide ring for hydride transfer. The catalytic residues identified in the yeast LYS1 structure are conserved and present in identical locations, Lys93 and His111 in this structure (Supplementary Figure 5) [27]. The adenosine diphosphate is only bound by Tyr284 and saccharopine itself. We previously speculated that the preference for NADPH over NADH was due to Arg267 making an ionic interaction with the 2’ phosphate [1]. Indeed, we observe this interaction but also note interaction with Ser266 and Thr224, as well as the amide backbone of Arg267 at the end of an α-helix. That most of the contacts to NADPH go to the 2’ phosphate explains the exquisite selectivity for NADPH that can drive the reaction in the forward direction. The saccharopine product is bound by arginines to its carboxylic acids, namely Arg146 and Arg39. The third carboxylic acid is coordinated by Gln116. Lys114 is close to the third carboxylic acid so it is possible that it contacts the lysine substrate, since we can only observe the product in this structure. Lastly, we note that while the LOR domain is quite rigid in our cryo-EM structures, there is a large conformational change upon substrate binding. A comparison between the new substrate bound structure and the previous apo structure revealed that the lobes close together upon substrate binding, with the loops and helices moving significantly closer from the left lobe with respect to the right lobe (Figure 2E). This suggests that significant motion is required for substrate binding and product release. In the structures of yeast LYS1, it was hypothesized [28] and then observed [27] that a salt bridge from Lys144 to Asp358 connects the two lobes of the enzyme and helps maintain a closed confirmation. These residues are conserved from yeast to human and indeed we see this salt bridge in the closed substrate bound complex but not in our previous apo crystal structure (Figure 2E).

### The effect of N-ethylmaleimide and its analogs on LOR

We have demonstrated that NEM can activate LOR activity through conjugate addition of Cys414 to the double bond of the maleimide in a Michael addition reaction [1]. The dose-response curves show an optimum, likely reflecting activation of LOR through C-S bond formation of Cys414 at low NEM concentrations, and inactivation of LOR due to additional unknown modifications at higher concentrations. To further study the mechanisms underlying these processes, we compared the effects of 12 maleimide analogs on LOR activity directly with NEM. The NEM analogs have different substituents attached to the nitrogen, but some have substitutions on the α,β-unsaturated carbonyl system. The ability of a maleimide to activate LOR may depend on its reactivity, which is determined by the physicochemical properties of these substitutions. These substitutions can also affect the binding affinity of the small molecule at the modification site. We observed that some analogs performed better than NEM, activating LOR at lower concentration and/or with a more pronounced and sustained activation. Others did not activate or produced pronounced inactivation (Supplementary Table 2 and Supplementary Figure 6A). As anticipated, the activation patterns were not only concentration, but also time-dependent (Supplementary Figure 6B).

**Figure 3.**
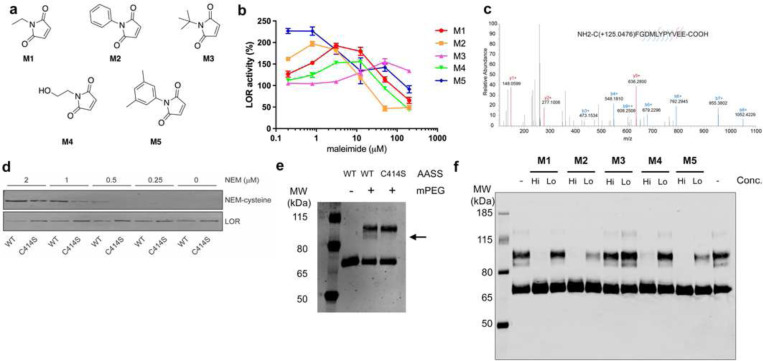
Maleimides’ ability to activate LOR reflects their reactivity toward Cys414. (A) Structures of 5 maleimides use for the studies described in this figure. (B) Effect of different concentrations of M1–5 on activity of the isolated LOR enzyme (n=4 for each concentration). (C) A representative MS/MS spectrum (HCD) of a NEM modified peptide. (D) Immunoblot of NEM-modified wild-type and p.C414S LOR detected with NEM-cysteine and His tag antibodies. Approximately 100 ng of protein was incubated with NEM at the indicated concentration for 10 minutes at room temperature before adding DTT and preparing the samples for immunoblotting. (E) Immunoblot of wild-type or C414S protein after labeling with mPEG 10K. Arrow indicates site of single C414 labeling. (F) Immunoblot of wild-type LOR pretreated for 10 minutes with M1–5 at a high (62.5 μM) and low (15.6 μM) concentration followed by mPEG labeling for 30 minutes then detection with 6X His Tag Polyclonal Antibody.

We next studied the reactivity of 5 selected maleimides in more detail, NEM itself (M1), N-phenylmaleimide (M2) and N-dimethylphenylmaleimide (M5), which activate LOR better than NEM, and N-*tert*-butylmaleimide (M3) and N-hydroxyethylmaleimide (M4), which produce lower activation of LOR than NEM (Figure 3A,B). We obtained a high-resolution crystal structure of LOR bound to M4 and M5, but observed no differences with our previously reported NEM and apo structure (data not shown). We observed no interaction of M5 with any of the LOR residues. We suspected, therefore, that the maleimides are activating the protein differentially based primarily on their reactivity with the protein and not by inducing different conformational changes.

To test this hypothesis, we first used proteomics to monitor the alkylation of LOR by NEM and other maleimides. Consistent with our prior work, Cys414 is the residue most efficiently labeled by NEM, but additional cysteine residues are alkylated as well (Figure 3C and Supplementary Table 3). We were able to detect alkylation of Cys414 by M3 and M4 confirming their reactivity. Technical challenges precluded the direct detection of alkylation of Cys414 by M2 and M5 most likely related to the hydrophobicity of the modified peptide. Using a monoclonal antibody recognizing NEM-modified cysteine residues in a sequence-independent manner [29], we observed that at low NEM concentrations the modification is undetectable in the C414S variant protein (Figure 3D), confirming that Cys414 is uniquely reactive.

To compare the reactivity of these selected maleimides, we then used maleimide polyethylene glycol (mPEG), which labels proteins with a predefined additional mass, 10 kDa with this reagent. We demonstrate that LOR is labeled by mPEG in a concentration-dependent manner revealing multiple modifications (Supplementary Figure 7). By using a C414S variant protein, we were able to identify a single modification for the Cys414 residue (Figure 3E), which is consistent with its high reactivity towards maleimides. We then compared the ability of the different maleimide analogs to compete with the mPEG labeling. M2 and M5 were more effective in competing with the mPEG labeling as compared to NEM. M4 was slightly less effective than NEM, whereas M3 could not prevent mPEG labeling at the same concentrations (Figure 3F). Therefore, we conclude that the reactivity of these maleimides correlates well with their ability to activate LOR through alkylation of the Cys414 residue.

### Mechanism of LOR activation by maleimides

**Figure 4.**
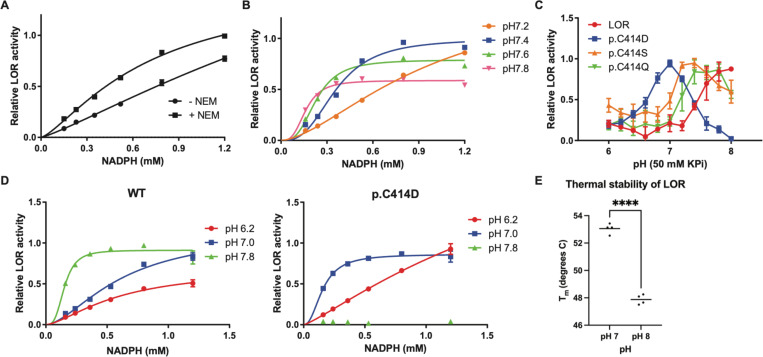
pH dependent changes in steady-state kinetics of LOR are mediated by Cys414. (A) Steady-state kinetic properties of LOR with and without alkylation by NEM (n=4 per condition). NADPH concentration was varied at fixed concentrations of lysine (15 mM) and 2-OG (1 mM). (B) Steady-state kinetic properties of LOR in KPi buffer of different pH (n=4 per condition). NADPH concentration was varied at fixed concentrations of lysine (15 mM) and 2-OG (1 mM). (C) Apparent pH optimum of LOR and selected Cys414 variants in KPi buffer of different pH under standard assay conditions (0.3 mM NADPH, 15 mM lysine and 1 mM 2-OG, n=4 per condition). (D) Steady-state kinetic properties of wild-type and C414D LOR in KPi buffer of different pH (n=4 per condition). NADPH concentration was varied at fixed concentrations of lysine (15 mM) and 2-OG (1 mM). For A-D, all error bars indicate SD. (E) Thermal stability of LOR at different pH (n=4 line shows the mean).

To determine the mechanism of LOR activation by maleimides, we determined the steady-state kinetic properties of LOR with and without alkylation by NEM. The most pertinent changes were observed for NADPH and not for lysine and 2-oxoglutarate, The progress curves for the different NADPH concentrations revealed multiple effects of the addition of NEM (Supplementary Figure 8A). Most prominently, at low NADPH concentration, the reaction rate is much higher in the NEM-modified LOR, indicating an increased affinity for this substrate. Indeed, using an allosteric sigmoidal model revealed decreased Khalf and Kprime values (Figure 4A, Supplementary Table 4). The progress curves, however, are not linear, showing a lag phase in the steady-state kinetics that is most pronounced in the non-modified LOR protein (Supplementary Figure 8A). Mechanistic reasons for such a lag phase include slow activation by the substrate (i.e., NADPH) or slow adaptation of the enzyme structure to the new reaction conditions (hysteresis) [30].. Mechanistically, these results illustrate that alkylation of Cys414 by NEM increased the affinity of LOR for the NADPH co-substrate, possibly through improving cooperativity.

We noted that the activation of LOR by NEM was dependent on pH of the potassium phosphate (KPi) assay buffer. Activation occurred at pH 7.2 and 7.4 and was absent at pH 7.6 and 7.8 (Supplementary Figure 8B). NEM reacts with the thiolate anion of Cys (Cys-), therefore activation is expected to be pH-dependent upon formation of the anion. In order to discriminate between the effects of pH and alkylation, we next restudied the pH optimum of LOR. In KPi buffer, LOR and full-length AASS (AASS-Myc-DDK in cell lysate) have a pH optimum around 7.8, with the standard assay pH of 7.4 being suboptimal (Supplementary Figure 8C). We then determined the steady-state kinetic properties of LOR at pH 7.2, 7.4, 7.6 and 7.8. Again, the most pertinent changes were observed for NADPH and not for lysine and 2-oxoglutarate. The progress curves revealed the most pronounced effect of pH on the LOR reaction rate at the lowest NADPH concentrations (Supplementary Figure 8D). LOR kinetics for NADPH changed markedly within this pH range showing a sharp increase in the Hill coefficient at higher pH (Supplementary Table 4). These results illustrate that the observed pH optimum of LOR is partly determined by cooperativity of NADPH binding (Figure 4B).

Since both the alkylation by NEM and pH appeared to affect NADPH binding, we next determined the effect of maleimide alkylation on the LOR pH optimum. NEM, but also M4, M2 and M12 shifted the pH optimum to a lower pH, consistent with the apparent activation under standard assay conditions of pH 7.4 (Supplementary Figure 8E). We next evaluated the effects of several Cys414 variants on the LOR pH optimum. Changing Cys414 to Ser, Gln or Asp lowered the optimum pH comparable to the effect of maleimide alkylations (Figure 4C). The effect of the Cys414Asp change was most pronounced with a clear pH optimum at 7, indicating an almost 1 log shift in pH optimum. At pH 7.8, the Asp414 variant appeared virtually inactive. Remarkably, Cys414 is not a conserved amino acid, and the mouse and zebrafish orthologs have a Tyr at this position (Supplementary Figure 5). We tested the pH optimum of the human Cys414Tyr variant and mouse LOR, which were both quite similar to human wild-type LOR (Supplementary Figure 9A).

Finally, we directly compared the steady-state kinetics of wild-type LOR and the Asp414 variant for the NADPH substrate at pH 6.2, 7.0 and 7.8 reflecting the shift in pH optimum (Supplementary Figure 9B, C). The Asp414 variant had notably higher cooperativity for NADPH at pH 7.0 when compared to the wild-type LOR (Supplementary Table 4). At pH 7.8, the Asp414 variant was inactive even at high NADPH concentrations (Figure 4D). We conclude that Cys414 in human LOR is important for cooperativity in NADPH binding in part explaining the pH optimum. To understand the changes that occur to the protein with these pH changes, we performed differential scanning fluorimetry on the LOR domain at different pH values. The protein showed a significant decrease in melting temperature at the higher pH value closer to the pH optimum, indicating that increasing flexibility of the protein is correlated with increased activity (Figure 4E). This change was less evident in DHTKD1, another enzyme in the pathway (Supplementary Figure 10).

### Structural dynamics of LOR induced by substitutions of Cys414

**Figure 5.**
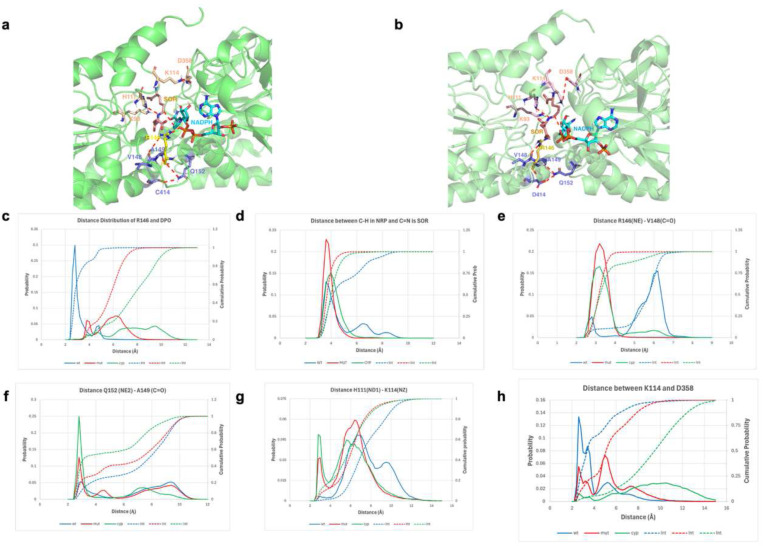
Representative snapshots for wild-type (Cys414) (A) and variant (Asp414) (B) LOR from MD simulations. The snapshots are the centers of the largest cluster (35% for wild-type, 34% for variant) out of 5 clusters obtained from a 2D RMSD of the trajectories. The broken red lines represent connections that are discussed in the text. The residues proximal to Cys414 in wild-type or to Asp414 in mutant, are colored ice blue. Arg146 is yellow, NADPH cyan, SOR orange and the residues interacting with SOR are colored in pink. (C-H). Probability distributions of distance between indicated residues from MD simulations of wild-type and Asp414 and CYP variants of LOR. Wild-type is colored in blue, Asp414 variant in red and CYP variant in green. The broken lines represent the cumulative probability.

To better understand how mutation or alkylation at the allosteric Cys414 residue could alter the kinetics of the enzyme, we performed simulations in which we mutated or alkylated the Cys414, modeled the oxidized saccharopine (SOR) based on the ternary complex in the cryo-EM structure, and looked at the key residues that interact with the substrates and connect to the allosteric site (Figure 5A and 5B). MMGBSA analysis shows that in the wild-type, the bridging diphosphate (DPO) is held by Arg146; this electrostatic interaction contributes more than 90% of the interaction energy with DPO (8.1 kcal/mol). However, in the Asp414 and CYP variants, this interaction diminishes to 2.0 and 1.7 kcal/mol, respectively. Arg146 now interacts with SOR and the interaction in the Asp414 and the CYP variants increases to 7.9 and 7.3 kcal/mol from 3.5 kcal/mol in the wild-type. Arg146 forms a close interaction with the NADPH most of the time in the wild-type simulations, but in the Asp414 and CYP variant simulations they are not within interacting distance (Figure 5C). The consequence of this shift is a stabilization of the SOR and an enhancement of its interaction with the nicotinamide ribose phosphate (NRP) portion of the NADPH. A critical distance is between the C4 atom in the dihydronicotinamide that carries two hydrogens and the imine bond produced by the conjugation of the terminal amine of lysine to the 2-position of glutarate (C6 in SOR), which must be in proximity for the hydride transfer to occur. From the distance distribution between C4 in NRP and C6 in SOR (Figure 5D), it is clear that in the wild-type only 67% of the C4-C6 distances are within hydride transfer. In the Asp414 and the CYP variants the distances reach 99.5% and 95%, respectively. The importance of this distribution is twofold. First, the proximity of the hydride donor to the enamine enhances the catalytic reaction. Furthermore, it may also increase the apparent affinity for NADPH.

Arg146 acts to organize the allosteric effect that travels from the site of residue 414 (Cys, Asp and the alkylated Cys derivatives) to the C-terminal helix. In the direction of residue 414, the Arg146-NE interacts with the carbonyl of Val148; in the wild-type the interaction is only 10%, often bridged by a water molecule (Figure 5E). In the Asp414 variant the interaction is 100% of the time and the bridging water disappears. The CYP variant maintains the interaction for 86% and the bridging water resides for only 6% of the time. Val148 and Ala149 are in a β-strand, which orients the carbonyl of Ala149 in the direction of residue 414. The distance, however, is too large for an interaction (>5.4 Å); the sidechain amide nitrogen of Gln152 acts as a bridge that depends on the nature of residue 414. In the wild-type, the interaction with the C=O of Ala149 persists for 25% of the time, while in the Asp414 variant it increases to 40% and in the CYP variant to 60% (Figure 5F). On the other side, Gln152 also interacts with residue 414 often with the help of a bridging water molecule; in the wild-type all the interactions are through a bridging water molecule present only 0.6% of the time. In the Asp414 variant, the total interactions represent 14%, of which 3% are direct H-bonds and 11% through a bridging water molecule (Supplementary Figure 11). In the CYP variant, the direct H-bond represents 22% and the water mediated only 5% (total interactions are 27%). Therefore, Gln152 is a critical residue that connects residue 414 with Arg146 in regulating the intensity of the allosteric effect observed in our studies. The interaction between CYP and Gln152 is striking compared to wild-type and Asp414 variant simulations. This shows that although they take a similar path, the allosteric effects observed have different mechanisms depending on the variations at residue 414.

The other interactions controlled by Arg146 are those with the various negatively charged groups of SOR. The Nη1 and Nη2 groups of the guanidinium in Arg146 interact with different carboxylates in SOR of the glutarate and the carboxylate of the conjugated lysine, not necessarily at the same time. In the wild-type, Nη1 forms an H-bond with O1 49.8% of the time while the H-bond to Nη2 is 45.3%. In the Asp414 variant, the H-bonds increase to 81.3 and 84.3%, respectively. In the CYP variant, the H-bonds to Nη1 and Nη2 are 76.0 and 91.2%, respectively. Another residue that forms H-bonds with the negative groups of SOR is Lys93. In the wild-type, the H-bonds sum up to 51.7%, in the Asp414 variant to 86.7% and in the CYP variant to 84.8%. The approach of His111 is in part caused by Lys114, which shows a lack of interaction in the wild-type protein (Figure 5G). Earlier, we discussed Lys114 as a latch that connects the two lobes. In the wild-type simulations, Lys114 interacts with Asp358 for 64% of the time (Figure 5H). This interaction progressively diminishes in the Asp414 variant to 36% and in the CYP variant to around 5%. The decrease in the interaction with Asp358 is correlated with the increase in the interaction with His111. The consequence of the disruption of the Lys114-Asp358 interaction determines the open and closed configuration of the enzyme. The wild-type enzyme therefore adopts a closed conformation much more frequently, while the variants have substantially more flexibility. Through all of these interactions, the residue at position 414 is able to affect the dynamics of the enzyme and favor a more active state, even though the residue is more than 12 Å away from the NADPH binding site.

### A screen reveals new metabolite inhibitors and activators of LOR

**Figure 6.**
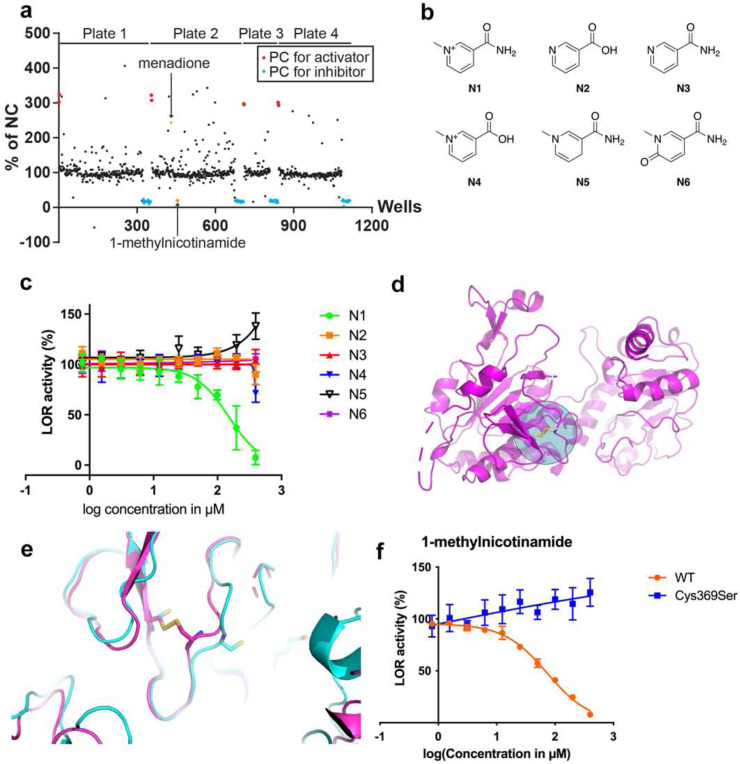
A high throughput screen of an endogenous metabolite library reveals inhibition of LOR by 1-MN^+^. (A) Overview of screening results with a set of positive controls (PC) for activation and inhibition for each of the 4 library plates. Each well is represented as the percentage activity of the negative control (NC, solvent). (B) Structures of 1-MN^+^ (N1) and tested analogs (N2–6). (C) IC_50_ curves for 1-MN^+^ and tested analogs. Compounds N1–4 and N6 were tested in quadruplicate, compound N5 in triplicate. All error bars indicate SD. IC_50_ for 1-MN^+^ was 146 μM. (D) Structure of LOR after incubation of 1-MN^+^ with the disulfide bridge indicated. (E) Close up of the disulfide bridge between Cys369 and Cys377. (F) IC_50_ curve for 1-MN^+^ in wild-type LOR and the C369S variant. Each condition was tested in quadruplicate. All error bars indicate SD. IC_50_ for 1-MN^+^ was 74 μM.

Since we had identified allosteric modulation of LOR activity, we hypothesized that there are endogenous metabolites that can mediate such regulation as demonstrated for many other enzymes. Indeed, L-2-hydroxyglutarate has been shown to inhibit LOR.[31] The inhibition by L-2-hydroxyglutarate was competitive with respect to 2-oxoglutarate (not shown) and relatively specific when compared to 3-hydroxyglutarate and D-2-hydroxyglutarate (Supplementary Figure 12A). To identify other novel endogenous metabolites capable of affecting LOR activity, we screened a 980-compound metabolite library (Supplementary Table 5). Based on the decrease in absorbance, we found 20 activating compounds (z-score >3) and 9 inhibiting compounds (z-score <−2) (Figure 6A, Supplementary Table 5). We noted multiple enzyme cofactors and polyamines in the group of top scoring activators. We repurchased 8 potential activators including riboflavin phosphate (FMN), methylcobalamin, menadione, cysteamine, phytosphingosine, pyrrole-2-carboxylic acid, spermine and spermidine. We also included putrescine, another polyamine, although this was not a hit molecule. Cysteamine, phytosphingosine and pyrrole-2-carboxylic acid did not replicate and therefore were considered false positives. FMN and methylcobalamin were unable to activate LOR. Incubations without enzyme suggest that these cofactors can oxidize NADPH non-enzymatically, which is supported by published work [32, 33]. We conclude that other hit co-factors such as flavin adenine dinucleotide and hydroxocobalamin are likely artefactual positives as well. The polyamines, spermine, spermidine and putrescine activated LOR with increasing chain-length leading to higher efficacy, but overall appeared less effective than in the screen (Supplementary Figure 12B). Menadione, a vitamin K precursor, consistently activated LOR (Supplementary Figure 12C). Menadione can arylate cellular nucleophiles such as cysteine [34]. This is reminiscent of the activation of LOR through alkylation of Cys414 by NEM. Indeed, the Cys414Tyr variant was not activated by menadione.

For the inhibitors, we repurchased 4-acetamidobutanoic acid (N-acetyl GABA), *trans*,*trans*-farnesol, γ--glutamylphenylalanine (γ-Glu-Phe), pyrogallol, 1-methylnicotinamide (1-MN^+^, TRIA-662) and neopterin. We also included spaglumic acid (N-acetylaspartylglutamate; NAAG), which were among the top scoring inhibitors. N-Acetyl GABA inhibited LOR, but with low potency (Supplementary Figure 12D). Pyrogallol and 1-MN^+^ had greater inhibitory activity. Pyrogallol, an organic reducing agent not known to be an endogenous metabolite, was not further studied. 1-MN^+^ is the first metabolite in the degradation of nicotinamide (vitamin B_3_), a precursor for NAD^+^. None of the other NAD^+^-related metabolites in the metabolite library displayed activity (Supplementary Table 5). To further confirm this observation, we compared the activities of niacin (**N2**), nicotinamide (**N3**), trigonelline (**N4**), 1-methyl-1,4-dihydronicotinamide (1-MNH, or “reduced 1-MN^+^, **N5**) and N-methyl-2-pyridone-5-carboxamide (2PY, **N6**) with 1-MN^+^ (**N1**) (Figure 6B). None of these metabolites was able to inhibit LOR, indicating that the organic cation and the amide groups are essential for activity (Figure 6C).

Although 1-MN^+^ is a simpler structure, it shares important features reminiscent of the product NADP^+^, i.e. a pyridinium ion comprising nicotinamide. The inhibition of 1-MN^+^, however, was not competitive with respect to NADPH, in contrast to NADP^+^ (Supplementary Figure 12E,F). To determine how 1-MN^+^ inhibits LOR, we attempted co-crystallization studies, but were unable to see any bound compound or obvious changes to the apo protein structure. We then used cryo-EM to obtain a complex of full-length AASS bound to 1-MN^+^, but also observed no density for bound compound. However, upon closer examination, we found that a loop near the active site flipped nearly 180° with a new disulfide bond formation observed between Cys369 and Cys377 (Figure 6D). We used multiple approaches to establish that this conformational change and the new disulfide bond are the mechanism of 1-MN^+^ inhibition. First, dithiothreitol was able to prevent inhibition of LOR by 1-MN^+^ (Supplementary Figure 12G), which is consistent with the notion that the disulfide formation is essential for inhibition. To formally test this, we mutated Cys369 into Ser. The Cys369Ser variant was resistant to LOR inhibition by 1-MN^+^ (Figure 6E). Thus, 1-MN^+^ does not appear to be mimicking the product NADP^+^ but rather induces a conformational change and irreversible disulfide bond formation based on proximity of the cysteines.

## Discussion

AASS initiates the lysine degradation pathway and catalyzes the first two enzymatic reactions. The first step is likely rate-limiting since NADPH serves as the co-substrate. It is therefore highly likely that lysine degradation is at least partially controlled at the level of AASS. Understanding how lysine degradation is regulated may provide novel therapeutic options for two disorders of lysine degradation that occur downstream of AASS, GA1 and PDE-ALDH7A1. Despite this, there are only a few studies on the regulation of lysine degradation, which have mainly focused on livestock since lysine is often the first or second limiting amino acid in animal feed [35–37]. Here, we report the cryo-EM structure of the full-length 400 kDa tetrameric AASS enzyme complex with substrates and products bound to both the LOR and SDH catalytic domains. The structure reveals a highly dynamic complex that can shuttle substrates and products between the domains connected by an alpha helix. In yeast, the two enzymatic activities are carried out by two separate proteins, but in higher eukaryotes they are carried out by a single bifunctional protein. From our structures, we observe the dynamic motion of the domains and speculate that this bifunctionality both increases efficiency and protects the first reaction product, saccharopine, from diffusing away from the enzyme.

Furthermore, through chemical screens, we identified allosteric compounds that modulate AASS activity. Serendipitously, we previously discovered that Cys414 can be modified by NEM to activate the enzyme [1]. Here, we show that changes at Cys414 cause large conformational changes that affect enzyme kinetics. Through a subsequent metabolite library screen, we discovered that 1-MN^+^ acts as an inhibitor of LOR. 1-MN^+^ is an endogenous metabolite produced by nicotinamide N-methyltransferase as part of NAD^+^ metabolism, specifically the irreversible degradation of nicotinamide. 1-MN^+^ is also known as TRIA-662 and shows antithrombotic and anti-inflammatory activities [38]. Cryo-EM studies showed that 1-MN^+^ induces the formation of a selective disulfide bond between cysteines 369 and 377 in the active site. We note that these cysteines are conserved and the formation of the disulfide bond may be a physiological inhibition mechanism. Although thoroughly validated as a hit molecule, 1-MN^+^ may not be an endogenous physiological ligand that induces the formation of this disulfide bond. Typical human plasma concentrations of 1-MN^+^ are 132 nmol/L [39, 40], which is much lower than the observed LOR IC_50_ values. One can speculate that mitochondrial oxidative stress conditions could oxidize these cysteines to a disulfide bond, thereby inactivating AASS and preserving NADPH for mitochondrial antioxidant processes. Combined, our findings illustrate that the LOR enzyme is highly dynamic and can be modulated at multiple sites through small molecules.

We observed that maleimides are able to activate LOR through alkylation of Cys414. The ability of a maleimide to activate LOR may depend on its reactivity, which is determined by the physico-chemical properties of the substitutions. The maleimides are reactive because of the α,β-unsaturated carbonyl system that makes them excellent Michael acceptors of nucleophiles including thiols such as that of cysteine. Substituents on the α,β-unsaturated carbonyl system or the nitrogen atom of the maleimide ring can influence the reactivity of the analogs in several ways. Electronic effects can be inferred based on the nature of the substituent and general principles of inductive and resonance effects. Electron-withdrawing groups can enhance reactivity towards the nucleophile and thus increase the rate of Michael addition which may be less specific for thiols. This might explain the pronounced inactivation of LOR by low concentrations of 3-bromo-1-phenyl-2,5-dihydro-1H-pyrrole-2,5-dione (M9) and some of the other inactivating maleimides (M10 and M13). Conversely, electron-donating groups on the nitrogen such as 1-(2-hydroxyethyl) or 1-phenyl or 1-(2-aminoethyl) (M4, M2, M5) can increase the electron density of the double bond, making it less electrophilic and reactive towards nucleophiles, potentially explaining a mild and predominant activating property, respectively. In addition to influencing reactivity, sterically bulky groups such as the N-*tert*-butyl group of N-*tert*-butylmaleimide (M3) reduced the reaction rate. Some substitutions may improve binding affinity through additional interactions at the modification site. Indeed, our work suggests that changing Cys414 into Asp creates two new H-bonds to neighboring residues, namely Trp153 and Gln152. Our crystal structures with CYN or CYP, however, did not find any evidence that the cysteine modification created any new interaction with neighboring residues. Further studies are needed to completely understand this effect. Screening of larger libraries of electrophilic fragments may lead to the identification of small molecules that interact at this site with more selectivity [41, 42]. Although this site is not conserved, our chemical probes indicate that it is a natural site to regulate the protein. We also note that this cysteine is uniquely reactive in the protein. It is possible that compounds with lower reactivity that also make additional protein interactions could be more selective.

More intriguing is the observation that mutation or alkylation of Cys414 has profound effects on the kinetics of the enzyme, despite the fact that the residue is more than 12 Å from the active site NADPH. The role of pH on the enzyme is quite complex, especially because even the substrates have several atoms with pKa values in the 6–8 pH range in addition to several catalytic residues. Therefore, to understand the effect of this residue we focused on the holistic changes of the LOR conformation that can be driven by these changes. The simulations show that changing Cys414 disrupts a latch-like interaction between Lys114 and Asp358. The large distance between residue 414 and the latch qualifies it as an allosteric effect, which changes the ensemble towards a wider range of conformations that is catalytically more active. This is supported by the thermal stability data in which the pH where the protein is more stable results in less activity. The allosteric path we were able to trace points to Arg146 as a critical residue that on the one hand organizes the residues proximal to residue 414 (Val148, Ala149 and Gln152) and on the other hand creates a connection between the substrate SOR and residues in its vicinity (Lys93, His111 and Lys114). The shift in the interaction of Arg146 with the DPO of NADPH to that with SOR, induced by the changes in residue 414, not only changes the remote latch, but also induces a more effective approach of the NADPH to the SOR enhancing the chance of a hydride transfer to the substrate. Furthermore, it creates the right environment for His111 to occupy a correct position to complete the reduction process. These allosteric changes enhance both the catalytic step as well as the affinity of NADPH. The molecular dynamics studies show how local changes to a protein can allosterically affect affinity for a substrate and thus activity through subtle effects propagated through the protein. It is important to view the effects of allosteric changes through dynamic changes in the protein, rather than individual sidechain motions. Intriguingly, variation at both Cys414 and pH of the reaction altered affinity for NADPH. From a more physiological perspective, one could envision that modulation of LOR activity may fine tune lysine degradation depending on mitochondrial redox state, substrate availability, or both.

The effects are likely amplified by the cooperativity of the tetrameric subunit. Nevertheless, it shows that subtle changes distal from the active site can have profound effects on the activity of an enzyme, particularly if they are located near the hinge of two lobes. Indeed, the key to AASS activity, like all enzymes, is to have dynamic flexibility to move between different conformations of the catalytic cycle. Small changes that affect flexibility, either through a disulfide bond formation induced by 1-MN^+^, or changes propagated by Cys414, have the ability to inhibit or reduce inhibition of the enzyme. This has large ramifications for designing inhibitor and activators of enzymes.

In conclusion, structural, pharmacological, biochemical and biophysical studies support the notion that AASS is likely to exert substantial control over the rate of lysine degradation. Further study of this enzyme is expected to lead to the discovery of new ways to modulate lysine degradation flux, which has the potential to impact treatment of disorders of lysine degradation.

## Supplementary Material

Supplementary Files

This is a list of supplementary files associated with this preprint. Click to download.

- LazarusAASSSupplementary2026.docx
